# Acceptance and Completion Rates of 3-Month Isoniazid-Rifampicin (3HR) Tuberculosis Preventive Treatment (TPT) Among Contacts of Bacteriologically Confirmed TB Patients—Patients’ and Healthcare Workers’ Perspectives

**DOI:** 10.3390/tropicalmed9120301

**Published:** 2024-12-07

**Authors:** Austin Ihesie, Ogoamaka Chukwuogo, Rupert Eneogu, Olugbenga Kayode Daniel, Aderonke Agbaje, Bethrand Odume, Debby Nongo, Charles Ohikhuai, Nera Kadiri-Eneh, Omosalewa Oyelaran, Victor Obianeri, Wayne Van Gemert, Enos Okumu Masini, Cleophas D’auvergne, Urhioke Ochuko, Chukwuma Anyaike, Sunday Olakunle Olarewaju

**Affiliations:** 1 United States Agency for International Development, Central Business District, Abuja 900211, Nigeria; aihesie@usaid.gov (A.I.); reneogu@usaid.gov (R.E.); dnongo@usaid.gov (D.N.); ooyelaran@usaid.gov (O.O.); vobianeri@usaid.gov (V.O.); 2 KNCV Nigeria, Central Business District, Abuja 900211, Nigeria; ochukwuogo@kncvnigeria.org (O.C.); bodume@kncvnigeria.org (B.O.); neneh@kncvnigeria.org (N.K.-E.); 3 Institute of Human Virology Nigeria (IHVN), IHVN Towers, C00 Emeritus Umaru Shehu Ave, Cadastral, Abuja 900108, Nigeria; odaniel@ihvnigeria.org (O.K.D.); aagbaje@ihvnigeria.org (A.A.); 4 Viamo Technologies Limited, Abuja 900108, Nigeria; charlesohikhuai@gmail.com; 5 Stop TB Partnership, 1218 Geneva, Switzerland; waynev@stoptb.org (W.V.G.); enosm@stoptb.org (E.O.M.); 6 United States Agency for International Development Global Health Bureau, Washington, DC 20004, USA; cdauvergne@usaid.gov; 7 Federal Ministry of Health, Abuja 900108, Nigeria; urhioke.ochuko@gmail.com (U.O.); chuxxanyaike@yahoo.com (C.A.); 8 Department of Community Medicine, Osun State University, Osogbo 230101, Nigeria

**Keywords:** tuberculosis, TB preventive treatment, contacts, Nigeria

## Abstract

Providing tuberculosis preventive treatment (TPT) to close contacts of persons with TB is a core strategy recommended by WHO for the prevention and control of TB. Nigeria rolled out the 3-month Isoniazid-Rifampicin (3HR) shorter regimen TPT as a pilot for use among eligible adult and child contacts. This study assesses acceptance and completion rates of 3HR TPT among contacts and determines the perspectives of healthcare workers (HCWs) and contacts on acceptance and completion of 3HR TPT in Nigeria. In this cross-sectional descriptive study using mixed methods, records of TPT-eligible clients were retrospectively reviewed, while 18 purposely selected HCWs and 18 contacts on 3HR were interviewed. Of the 30,012 eligible contacts, 12,040 (40.1%) were initiated on TPT. Among these, 8213 (68%) were enrolled on 3HR, and 6972 (84.7%) of them completed treatment. Perceived facilitators include belief in its effectiveness, training among HCWs, and a good understanding of TPT from HCW counseling sessions. Barriers reported were linked to stockouts, misconceptions about side effects, non-disclosures, and disincentive follow-up strategies. The acceptance and completion rate for 3HR TPT was good. Scaling up 3HR TPT will require redesigning policies towards addressing identified barriers and utilizing interventions linked to capabilities, opportunities, and motivations among contacts of TB patients and HCWs.

## 1. Introduction

Every year, about 10 million people develop TB, and it is the 2nd leading cause of death worldwide. In 2022, Nigeria ranked 6th among eight countries, globally accounting for two-thirds of the pandemic [1]. Latent TB infection (LTBI) refers to a state of persistent immune response to stimulation by MTB antigens without a clinical manifestation of active TB, and reports show that about 25% of the global population have latent TB infection [1,2]. Tuberculosis preventive treatment (TPT) is the intake of one or more anti-TB drugs by people with latent infection to prevent the progression of LTBI to active TB disease and also to prevent TB infection after exposure to a TB case [2].

In Nigeria, the national TB guidelines recommend six months of isoniazid (6H), three months of Isoniazid and Rifampicin (3HR), three months of Isoniazid and Rifapentine (3HP), and one month of Isoniazid and Rifapentine (1HP) as regimen options for use as TPT for adults and children at increased risk of exposure and progression to active TB [3]. The guideline, however, prioritizes adult and child household contacts of an index TB case and persons living with HIV (PLHIV) as high-risk groups for the programmatic intervention of TPT in the country [3,4]. Eligibility for TPT by Nigeria guidelines is for contacts of all ages without active TB disease. In order to rule out active TB disease, symptomatic contacts irrespective of age would undergo further evaluation procedures for TB, while the guideline recommends LTBI screening for HIV negative asymptomatic contacts above 5 years before being placed on TPT. However, due to resource constraints, the quantiferon assay tests are unavailable for use in the TB program [3].

Previous studies in settings around the world have shown various levels of acceptance (39–91%) and completion (29–90%) of the WHO-recommended treatments for LTBI [5,6,7,8]. The variations observed in the acceptance and completion of TPT may be due to the challenge of convincing patients without any symptoms of TB that they are infected and at risk of developing the disease. In addition, healthcare workers (HCWs) had reservations about deciding to place a healthy person on the TPT [9].

Other factors influencing the acceptance and completion of TPT were the inconsistent supply of TPT medications, the health education and counseling of eligible contacts, fear of adverse drug reactions, pill burden, and a lack of an integrated monitoring and evaluation system for TPT [10,11]. These may continue to threaten TB elimination in the face of the already existing public health challenge of non-adherence to anti-TB medication in the past years. Similarly, in a systematic review by Szkwarko et al. (2017), the authors identified issues with health system infrastructure, incorrect attitudes and perceptions, and competing priorities as part of contributory factors [12].

Among the TPT recommended in Nigeria, Isoniazid has been the only regimen available for use up until 2022, when 3HR was introduced in-country through the USAID/Stop TB Partnership’s introducing New Tools Project. Compared to the conventional 6 month Isoniazid monotherapy, 3HR is a shorter, more tolerable regimen. By making it available to all age groups, the burden of TPT on patients and healthcare systems may be lessened, and treatment completion rates may be increased.

This study assessed the acceptance and completion rates of 3HR TPT among contacts (0–14 years and >15 years) of persons with bacteriologically confirmed TB reported between April 2022 and September 2022 and also sought the perspectives of HCWs’ and eligible contacts of TPT on the uptake of 3HR TPT in healthcare facilities in Nigeria.

It is expected that the findings will help develop context-specific strategies, guidelines, and policies to bridge the TPT gap among the growing population of eligible clients not on TPT, thereby contributing to achieving the goal of the WHO End TB strategy.

## 2. Methodology

### 2.1. Study Design and Settings

A mixed-method cross-sectional study design was employed. The quantitative aspect involved collection of secondary data from patients’ hospital records on TPT, while the qualitative aspect involved in-depth interviews of HCWs providing 3HR TPT and contacts. Public and private health facilities that offer directly observed treatment short course (DOTS) for tuberculosis and 3HR TPT in the 18 states (Akwa Ibom, Anambra, Bauchi, Benue, Cross River, Delta, Imo, Kano, Kaduna, Katsina, Lagos, Nasarawa, Ogun, Osun, Oyo, Plateau, Rivers, and Taraba) implementing the USAID TB LON project in Nigeria were included as study sites.

### 2.2. Study Population and Sample Size

The study population included consenting HCWs in private and public facilities within the 18 TB-LON-supported states working in TB DOTS units that directly support TB patient management, TB contact investigation, administration, and management of TPT. In addition, contacts of persons with bacteriologically diagnosed TB who were enrolled on 3HR TPT and whose data were appropriately recorded and reported in the contact management register of 3HR TPT implementing facilities were included in this study. Non-consenting HCWs and contacts with incomplete data were excluded from the study.

For the qualitative aspect of the study, one HCW involved in the administration and management of 3HR for TPT and one contact (>15 years) who was placed on 3HR TPT were purposely selected from each of the 18 states involved in the study. However, for the quantitative component of the study, data were collated on the total contact investigation cascade for all facilities implementing 3HR TPT in the 18 TB LON project states. The data collection officer obtained information and ensured proper documentation of the consent forms for all respondents interviewed.

### 2.3. Data Collection Procedure

A data abstraction tool (Supplementary Materials) was utilized to gather quantitative data from hospital records of facilities implementing 3HR for TPT implementing 3HR for TPT in a longitudinal study for TB contacts enrollees between April 2022 to September 2022. This included information on indicators related to contact tracing cascade indicators data. A qualitative approach involved conducting in-depth interviews with contacts who received 3HR and HCWs to assess their perspectives and opinions of factors influencing contacts’ and HCWs’ acceptance and completion of the 3HR TPT. The interview guides were adapted from a similar study conducted in Uganda [13]. The research coordinator and technical leads provided one-day training session to eighteen professionals who were selected as data enumerators and had similar experience in conduct of studies related to TB activities. They were familiarized during 1-day training with how to conduct and audio-record interviews using pretested discussion guides among TB contacts (greater than 15 years) and HCWs as well as extraction of relevant indicators using contact management register. Audio-recorded information was later transcribed, translated into English as needed, and reviewed for accuracy and completeness by bilingual research staff. Information gathered using TB contact management register contained records of individual enrollees during the period of April 2022 to September 2022 regarding relevant indicators at various points in the TB care cascade. These data were collected longitudinally. Household contacts (individuals living in same household) of TB cases, i.e., confirmed by microbiological testing, had TB symptom assessment by contact tracers at home while those with symptoms had sputum test and chest X-ray performed within hospital settings to rule out active TB. Child (0–14 years) and adult (greater than 15 years) household contacts without active TB were placed on 3HR TPT in TB treatment facilities with regular follow-up measures implemented to ensure adherence and treatment completion. The study covered 138 facilities offering 3HR TPT, with 87 primary, 51 secondary, and 10 tertiary, respectively. Mentorship and supervisory visits were provided on weekly basis during the survey period to ensure compliance with the study protocols and regulations approved for the study.

### 2.4. Ethical Consideration

Ethical approval for this study was obtained from the National Health Research Ethics Committee of Nigeria (NHREC), and number: NHREC/01/01/2007 was assigned. Written/signed consent was also obtained before participation in the study concerning the qualitative technique.

In accordance with authority and permission granted by state and local government TB program managers, trained research assistants were utilized to gather data on 3HR TPT from TB contacts and to acquire information from pre-existing health records. Personal identification data were not collected during the process.

### 2.5. Data Analysis

Data collected were anonymized, and strict confidentiality was ensured. Quantitative data were analyzed using SPSS for Windows 10 version 25 statistical software (New York, NY, USA). Data were checked for completeness and accuracy through proof reading while entering the data into the software. Descriptive statistics (frequencies and percentages) were employed to generate tables.

The following variables were considered in the quantitative analysis concerning TB contact management cascade:

Number of Bacteriologically confirmed Index Patients Contact Traced: This is the total number of bacteriological index patients diagnosed whose contacts were traced.

Number of Contacts identified: This is the total number of contacts met in the household of bacteriologically confirmed index cases visited.

Number of Contacts screened for TB: This is the total number of contacts screened for TB symptoms among contact person met in household of bacteriologically confirmed index cases.

Number of Contacts presumptive for TB: This is the total number of contacts with symptoms of TB among all contacts met in the household of bacteriologically confirmed index cases that were symptomatic for active TB.

Number of Contacts Diagnosed with TB: This is the total number of presumptive contacts diagnosed as TB case after evaluation.

Number of Contacts Eligible for TPT: This is the total number of contacts with no active TB living with index TB cases that are eligible for TPT.

Number of Contacts Initiated on TPT: This is total number of contacts placed on TPT regimen among eligible.

Number of Contacts Enrolled on 3HR Regimen: This is total number placed on 3HR TPT among eligible.

Treatment Outcome of Contacts Placed on 3HR TPT:

(a) 3HR TPT Treatment completed: This is the total number of TB contacts placed on 3HR TPT who successfully complete 3HR TPT prophylaxis among total number of TB contacts started on 3HR TPT. It is usually expressed in proportion where the numerator is the total number of TB contacts placed on 3HR TPT who successfully complete prophylaxis and the denominator is the total number of TB contacts started on 3HR TPT over the same period.

(b) 3HR TPT Treatment not evaluated: This is the total number of TB contacts placed on 3HR TPT whose outcome unknown among total number of TB contacts started on 3HR. It is usually expressed in proportion with numerator as total number of TB contacts placed on 3HR TPT whose outcome is unknown while denominator is the total number of TB contacts started on 3HR TPT over the same period.

(c) 3HR TPT developed TB: This is the total number of TB contacts placed on 3HR TPT who developed tuberculosis while on treatment among total number of TB contacts started on 3HR TPT in same period. It is usually expressed in proportion with numerator as total number of TB contacts placed on 3HR TPT who developed TB while on treatment while denominator is the total number of TB contacts started on 3HR TPT over the same period.

(d) 3HR TPT loss to follow-up: This is the total number of TB contacts placed on 3HR TPT that were lost to follow-up while on 3HR TPT treatment among total number of TB contacts started on 3HR TPT in same period. It is usually expressed in proportion with numerator as total number of TB contacts placed on 3HR TPT lost to follow-up while on treatment while denominator is the total number of TB contacts started on 3HR TPT over the same period.

(e) 3HR TPT treatment interrupted: This is the total number of TB contacts placed on 3HR TPT who interrupted treatment among total number of TB contacts started on 3HR TPT in same period. It is usually expressed in proportion with numerator as total number of TB contacts placed on 3HR TPT who interrupted treatment while on treatment while denominator is the total number of TB contacts started on 3HR TPT over the same period.

Grounded theory was used during the qualitative analysis because it enabled the formulation of a new, process-oriented theory that takes into account the actual experiences of both HCWs and TB contacts as well as enhanced development of practical ideas for enhancing TPT uptake and completion in healthcare settings [14]. Two investigators developed codes after transcription based on the original terms used and matched. The transcripts were analyzed thematically by categorizing them per the specific objective.

## 3. Result

Data collated from the TB contact management register for all facilities implementing 3HR TPT in the 18 TB LON project states showed that a total of 7796 persons diagnosed with bacteriological confirmed pulmonary TB between April 2022 and September 2022 had their contacts traced. There were 33,343 contacts identified, of which 31,982 (96%) were screened for tuberculosis symptoms and 10,110 (33%) identified as presumptive. Tuberculosis was confirmed in 1113 (11%). Of the 30,012 eligible for TPT, 12,040 (40.1%) were initiated on TPT, out of which 8213 (68%) enrolled on the 3HR regimen while 3827 (32%) enrolled on the 6H. The majority of enrollees on 3HR were female 4436 (54%) and more than 15 years old, i.e., 5600 (68%). Also, 857 (2.7%) among 30,869 identified contacts without TB were not initiated, as they were ineligible.

**Figure 1:**
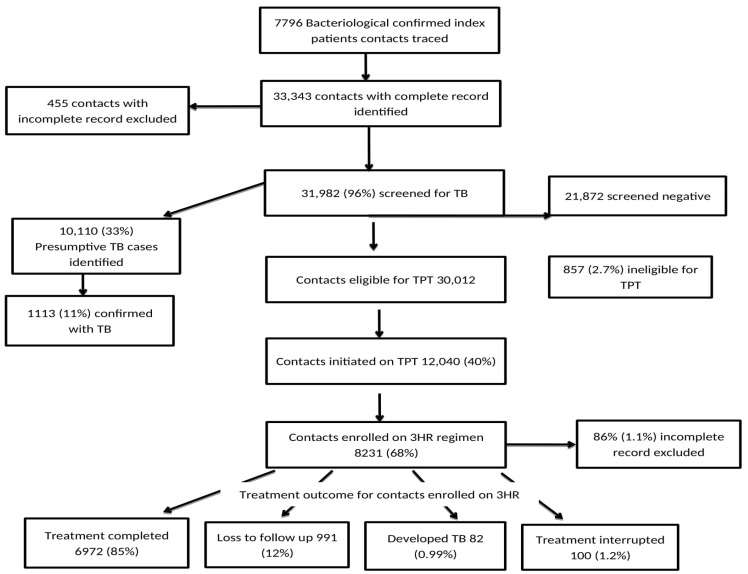
Acceptance and completion of 3HR TPT use by eligible contacts in Nigeria from April to September 2022.

In terms of treatment outcomes for the 8213 clients enrolled on 3HR, 6972 (84.7%) completed treatment, 991 (12.1%) were lost to follow-up, 82 (1%) interrupted treatment, and 100 (1.2%) developed active TB (Figure 1).

A total of 18 HCWs and 18 contacts who were on 3HR TPT were interviewed. Among the HCWs, the majority were within 25–46 years (46%), were females (61.1%) having tertiary education (94.4%), were mainly DOTS officers (55.6%), and worked with public-secondary/public-primary health facilities (38.9%). (Table 1) Among the contacts interviewed, the majority were greater than 30 years old (76.8%), were females (61.1%), had tertiary education (44.4%), and lived less than 1 km from the facility (61.1%). However, in terms of employment, only 27.8% were unemployed (Table 2).

### 3.1. Healthcare Workers and Contacts Reported Facilitators and Barriers to Acceptance and Completion of 3HR TPT

#### 3.1.1. Healthcare Workers Reported Facilitators

Healthcare workers (HCWs) were comfortable offering 3HR to eligible contacts and encouraged the use of the shorter regimen TPT medicine. Their views illustrated a belief in the safety and effectiveness of the drug in preventing users from getting infected with TB.

*3HR TPT is very effective. We don’t receive any negative feedback from most of the people on the drug.*

(Akwa Ibom HCW P1)

*We encourage contacts to go on the (shorter) 3HR for three months in order to protect themselves*

(Plateau HCW P1)

*It is very good, safe, and has no associated side effects.*

(Benue HCW P2)

*It has a shorter duration, safe and good medication for relatives*

(Anambra, HCW P1)

During clinic visits, HCWs allay myths and misconceptions concerning 3HR TPT among contacts by providing further counseling on drug safety and the associated benefits, emphasizing the need to come for follow-up monthly clinic visits and explaining common side effects and their management measures.

*We normally counsel and encourage them on how to prevent TB anywhere they find themselves, using nose masks and other preventive measures.*

(Akwa Ibom HCW P2)

*It is very effective, just for 3 months usage compared to 6 months isoniazid*

(Kaduna HCW P1)

Finally, HCWs believed effective communication during counseling sessions and taking time to explain to each patient the safety and benefits of 3HR TPT drugs, highlighting their shorter duration compared to Isoniazid, encourages clients to accept the 3HR TPT.

#### 3.1.2. Healthcare Workers Reported Barriers; This Is Classified into Two Categories

- HCW reported Barriers experienced by TB contacts

Almost all HCWs interviewed perceived inadequate knowledge of the safety and effectiveness of 3HR TPT despite persuasion in clinical settings as one key factor hindering acceptance and completion, with some clients wondering why they need to take the drugs when they do not feel sick.

*Some of them will directly tell you that they will not take the drug, but we do find a way to convince them to take it by making them understand that the drug they are to take is not for treating TB but for prevention against TB.*

(Bauchi HCW P2)

*They (Contacts) usually ask why they need to take drugs when they are not sick*

(Rivers HCWs)

Another major barrier elicited from the interview of HCWs was the challenge of access by contacts to pick up drugs at the facility due to their limited ability to fund transportation to the health facility for enrollment and subsequent follow-up visits.

*The issue here is finance; it is either they are too far from the clinic, or they have a financial problem*

(Anambra)

*The barrier there can be the distance because some of them (contacts) will ask or how long will I be coming here as I’m living so far away? We have a lot of people defaulting because of the challenge of coming*

(Plateau HCW P1)

b. HCW reported Barriers experienced within Healthcare system

Furthermore, other perceived barriers highlighted by HCWs included fear of side-effects and challenges of drug stockout and timely supply.

*Sometimes, we experience stockout of TPT, and we keep calling so we can get the supply, but we don’t receive them on time.*

(Lagos HCW P2)

*Some get worried about taking the drugs every day and the possible side effects.*

(Rivers HCW P1)

#### 3.1.3. Contacts Reported Facilitators

Almost all the respondents had a good understanding of tuberculosis, symptoms, and the mode of transmission as well as the benefits of 3HR TPT in protecting contacts from developing active tuberculosis. This information was received majorly during counseling sessions by HCWs during clinic visitations. They believed that SMS reminders would be useful mechanisms if put in place to ensure drug adherence within the three-month treatment period.

*It (3HR-TPT) is meant to protect yourself since we know we have someone who is infected. We don’t want to be infected, so we are taking it. If you are not taking the drugs as prescribed, you will contact TB. *

(47 years old male, Lagos-CTT1)

*If I use my drugs, the chances of me getting infected with TB will be very minimal. *

(Kastina-CTT 2)

*The medication is meant to prevent me from contracting TB, and therefore, I always ensure I take it all the time. *

(Rivers-CTT 1)

#### 3.1.4. Contacts Reported Barriers

About 39% of the contacts interviewed lived more than 1 km from the health facility where they received care, and the majority reported that the high cost of transportation was a major challenge to them accessing care. Some of the respondents expressed their preference for a home delivery of TPT medications or having their transportation bills subsidized by the government when they come for monthly refills.

*Home delivery will be prefered by me *

(Akwa Ibom–CTT 2)

*Support for transportation by Government will be needed *

(Lagos-CTT 2)

*“It is difficult to get transportation. My house is about 15 km away from the health center. It isn’t easy since we are far from the clinic”. *

(Benue-CTT 2)

Concern about stigma associated with Tuberculosis

The following questions and concerns are likely to arise among family members and friends if someone is known to be taking TB prevention medication:

*Why are you taking this drugs, are you infected? *

(47 years old male, Lagos)

*If people close to me get to know they will ask me if I am TB positive. *

(Kastina)

*“Yes, it will affect my relationships. If they know, they won’t like to associate with me again since it is a disease that people are scared of”. *

(Rivers CTB)

*“I don’t have anyone who knows what drug I am taking”.*

(Nassarawa CTB)

*“At first, we had some mixed feelings and challenges like dizziness”. *

(Kano-CTB)

*“I heard someone complaining about headaches and also excess periods, but my experience was fine”. *

(Plateau-CTB)

Tuberculosis is a stigmatized disease, particularly in communities with myths and misconceptions concerning etiology and disease transmission. Due to stigma, many respondents declined to disclose their status to family members, as they reported this would disrupt their relationship with them.

### 3.2. Options for Behavioral Diagnosis and Interventions Options

We examined behavioral determinants among household contacts of bacteriologically confirmed TB clients and HCWs using the COM-B model, a popular framework for changing behaviors that aims to explain and influence human behavior. The acronym COM-B represents the concepts of capability, opportunity, and motivation, which when combined, produce behavior [15]. Interventions can be more precisely tailored to the specific needs and barriers encountered when accepting and completing TB preventive treatment. This is broken down into three categories, capabilities, opportunities, and motivations, as shown in Table 3 (facilitators) and Table 4 (barriers), respectively.

The COM-B model was used to analyze the factors that facilitate people’s capability, opportunity, and motivation to accept and complete 3HR preventive therapy against tuberculosis (TB), which were outlined below according to Table 3.

Psychological and Physical Capabilities: This includes understanding the TB mode of transmission and the risk of contracting the infection on the part of household contacts. In addition, the belief in 3HR TPT effectiveness based on experience and training received was seen by HCWs as contributors to the acceptance and completion of 3HR TPT.

Physical and Social Opportunities: Supportive features include the availability of job aids together with counseling sessions in clinics and follow-up visits as well as training opportunities.

Motivation: This involves perceived benefits of 3HR based on previous training and experience by HCWs as well as allaying the fear of contacting a tuberculosis infection using 3HR preventive therapy regarding barriers in Table 4 the issues identified were highlighted below.

Psychological and Physical Capabilities: Low health literacy on the effectiveness and safety of 3HR TPT on the part of household contacts of bacteriological confirmed TB patients as well as the unavailability of 3HR TPT medication, a lack of incentives for telephone calls, and a lack of home visitation were seen on the part of HCWs as barriers.

Physical and Social Opportunities: Numerous factors, including the non-disclosure of the usage of 3HR TPT to family members due to stigmatization that can hinder receiving needed assistance from friends and family members on the part of household contacts as well as the location of facilities, were seen as barriers affecting the acceptance and completion of TB preventive therapy.

Motivation: Misconception about the effectiveness of 3HR among TB contacts as well as potential side effects of 3HR TPT as documented in in-depth interviews with the contact of bacteriologically positive TB clients and HCWs, respectively, will make individuals less motivated about accepting and completing 3HR TPT.

In the context of the COM-B model’s behavioral determinants, connecting the behavioral diagnosis obtained using the COM-B model to the developed framework would allow for prospective interventions to serve as appropriate functions to address the indicated barriers and facilitators, which in turn could improve the acceptability and completion of 3HR TPT among contacts of TB patients [15].

Furthermore, Table 5 summarizes potential interventions suggested by the researcher team to address the barriers taking into account the facilitators identified.

## 4. Discussion

This study was conducted to identify potential barriers and facilitators of the acceptance and completion of 3HR TPT, as perceived by contacts of persons diagnosed with TB and health workers in Nigeria using both quantitative and qualitative research methods to better inform the development of effective implementation programs concerning 3HR TPT in Nigeria. Quantitative data collated through a review of the existing record from the TB contact management register showed that 12,040 (40%) of the eligible contacts were initiated on TPT, with 8231 (68%) of the enrollees placed on the 3HR TPT regimen. Findings from the qualitative interviews provide some insight into some reasons for the low TPT enrollment, and they include the challenge of cost of transportation to access TPT care by contacts and a preference by contacts for a home-based care approach, where stigmatization, follow-up visits, and problems with care access are all addressed.

Our study showed that 32% of the contacts enrolled on 3HR were children < 15 years. Although this is lower than the proportion of children (42%) that make up Nigeria’s estimated population, it also indicates that children make up a significant proportion of the household in Nigeria and need to be prioritized, as they are at an increased risk when in contact with a household member with TB [16].

The TPT initiation rate of 40% reported by this study is lower than the rates reported from a study in Ethiopia, which reported 64.3% [17]. This study, however, reports a high completion rate of 85% for 3HR TPT among contacts, although this was still lower compared to findings from an Eswatini study (98%) by Kay et al. in 2023 [8]. The reason for this may be due to differences in settings where the study was conducted. Although the treatment completion rate on 6H could not be assessed within the period of the study, higher percentages reported for 3HR could be corroborated with qualitative interview findings among HCWs, where respondents reported a preference and tolerance to 3HR with no negative feedback.

This study’s qualitative survey showed that the acceptance and completion of 3HR TPT can be affected by misunderstandings of TPT’s preventive role among HCWs and contacts of persons with TB. Similar findings were reported by Mindachew et al. and Jacobson et al. among people living with HIV/AIDs using six months of isoniazid preventive therapy [18,19]. The result is also consistent with findings of cross-sectional study conducted in Malawi among child contacts by Triasih et al. in 2016 [20].

HCWs in this study were comfortable using 3HR TPT based on experience, the training received, and the shorter duration of treatment. They believed in its safety and benefits in preventing active TB among under five and adult contacts. The evidence of this is shown in the documentation practices based on quantitative data, where more than half of TPT enrollees (68%) were placed on the 3HR TPT regimen. This may be due to preferences for the 3HR TPT regimen supplied to the health facilities and, hence, a reason why a higher proportion of eligible contacts were enrolled on 3HR TPT [21].

During in-depth interview of contacts, it was elicited that poor knowledge of the benefits of 3HR and fear of 3HR TPT side effects were among issues that may prevent eligible contacts from accepting and completing 3HR TPT. It is plausible that the reluctance from contacts accepting TPT may also be due to the lack of TB infection testing, which could persuade clients to initiate TPT.

Transportation costs and the unavailability or stockout of the 3HR TPT regimen in some facilities have also been mentioned by some HCWs as barriers in this study. Furthermore, research on TPT conducted in Malawi and Indonesia reported on this [22,23]. In a comparable study conducted in South Eastern Asia [24] and India [25], stockouts of TPT were identified as a hindrance to TPT uptake.

This could be addressed through refresher trainings on logistics regarding the quantification and forecasting of 3HR TPT medicines among HCWs coupled with follow-up visitations.

When asked on challenges regarding health system infrastructure, HCWs further brought up issues related to the timely delivery and stockout of 3HR TPT drugs.

There are many mitigation strategies that will increase the use of 3HR TPT. One of these strategies is the use of community enrollment or home delivery as a substitute for facility-based 3HR [26], while enablers ranging from providing transport incentives for HCWs conducting home visits, telephone calls, and reminder text messages to providing transport incentives for contacts living in distant locations will help in improving the acceptance and completion of 3HR TPT services. Likewise, continuous education through counseling by HCWs on 3HR TPT before initiation and during follow-up monthly refills and refresher training among HCWs on 3HR TPT using updated guidelines will help in addressing issues related to safety, ignorance, and misconception regarding the effectiveness of 3HR TPT therapy identified among contacts of TB patients. Healthcare workers can leverage follow-up monthly visitation to persuade contacts to ensure acceptability and completion.

In conclusion, acceptance was low but the preference for the shorter regimen 3HR was remarkable, with 68% of patients initiated on 3HR compared with other similar study findings [8]. Moreover, the scale-up of 3HR TPT will require updating existing policies and guidelines toward addressing barriers and utilizing differentiated care models centered on community-based TPT delivery, where interventions are linked to capabilities, opportunities, and motivations among contacts and HCWs reported during the survey.

There were some limitations encountered while conducting this study. First, some data extracted using existing TB contact management registers were incomplete; these were excluded during the analysis process for accuracy and consistency. Additionally, a proportion of clients who refused treatment during the study period were not included in the cascade. In addition, only 18 HCWs and 18 TB contacts (who were >15 years) were purposively selected for the qualitative technique of data collection.

However, the mixed methodology and sampling approach applied in the study are significant strengths that validate the findings from the study.

## Figures and Tables

**Table 1:** Socio-demographic characteristics of HCWs.

Variable	Frequency (*n* = 18)	Percentage
Age group		
25–35 years	8	44.4
36–46 years	8	44.4
>46 years	2	11.1
Gender		
Male	7	38.9
Female	11	61.1
Level of education		
Secondary	1	5.6
Tertiary	17	94.4
Position at health facility		
Others	8	44.4
DOT officer	10	55.6
Type of facility		
Public-Tertiary health facility	4	22.2
Public-secondary health facility	7	38.9
Public-primary health facility	7	38.9

**Table 2:** Socio-demographic characteristics of TB contacts interviewed.

Variable	Frequency (*n* = 18)	Percentage
Gender		
Male	7	38.9
Female	11	61.1
level of education		
Primary	3	16.7
Secondary	7	38.9
Tertiary	8	44.4
Age group		
20–30 years	4	22.2
>30 years	14	77.8
Occupation		
Trading	2	11.1
Business woman	3	16.7
Teaching	1	5.6
Student	1	5.6
Civil servant	1	5.6
Self-employed	4	22.2
Unemployed	5	27.8
Farming	1	5.6
Distance from facility		
less than 1 km	11	61.1
>1 km	7	38.9

**Table 3:** Behavioral determinants of acceptance and compliance of 3HR TPT among contacts of TB patients and healthcare workers in Nigeria—Facilitators.

Behavior Determinants (Facilitators)	Healthcare Workers	Household TB Contact on 3HR Treatment
Capabilities	Belief on 3HR TPT effectiveness based on experience and training received	Understanding TB mode of transmission and risk of contracting infection
Opportunities	Training opportunities and availability of job aids	Counseling session during clinic and follow-up visits
Motivation	Perceived benefits of 3HR based on previous training and experience	Fear of contacting Tuberculosis

**Table 4:** Behavioral determinants of acceptance and compliance of 3HR TPT among contacts of TB patients and Healthcare workers in Nigeria—Barriers.

Behavior Determinants (Barriers)	Healthcare Workers	Household Contact on 3HR Treatment
Capabilities	Stockout of 3HR TPT, lack of incentives for telephone calls and home visitation to TB contacts	Inadequate knowledge of 3HR TPT effectiveness and safety
Opportunities	Location/proximity of facility	Non-disclosure of usage of 3HR TPT to family members
Motivation	Misconception about effectiveness of 3HR among TB contacts	Misconceptions about potential side effects of 3HR TPT

**Table 5:** Investigator-identified intervention functions targeting identified barriers and facilitators as defined in the behavior change wheel framework.

Intervention Function	Potential Interventions
Education	1. Intensify 3HR TPT counseling sessions before initiation and during treatment among TB contacts 2. Refresh training among HCWs on 3HR TPT using updated guidelines
Persuasion	Use monthly monitoring visits for proper counseling
Refining 3HR TPT service delivery	1. Provide home delivery of 3HR TPT services
Enablement	1. Provide incentives for HCWs for home visits, telephone calls, and reminder text messages 2. Provide transport incentives for contacts living in far locations.

## Data Availability

The datasets generated and analyzed during the current study are not publicly available, but are available from the corresponding author upon reasonable request.

## Supplementary Materials

The following supporting information can be downloaded at: https://www.mdpi.com/article/10.3390/tropicalmed9120301/s1, Data extraction tool.
