# Supplementary material for: Acceptance and Completion Rates of 3-Month Isoniazid-Rifampicin (3HR) Tuberculosis Preventive Treatment (TPT) Among Contacts of Bacteriologically Confirmed TB Patients—Patients’ and Healthcare Workers’ Perspectives
Source: Trop Med Infect Dis. 2024 Dec 7;9(12):301. doi: 10.3390/tropicalmed9120301 (PMC11679968; doi:10.3390/tropicalmed9120301)
Supplement: Supplementary file 1 [file tropicalmed-09-00301-s001.zip › tropicalmed-3200111-supplementary.pdf]

Data Extraction tool from contact management register (April 2022 – September 2022)

S/N	Variable Name	0-14 (M)	0-14 (F)	≥ 15 (M)	≥ 15 (F)
1.	# of index patients contact traced				
2.	# of contacts identified				
4	# of contacts screened for TB				
5	# of contacts presumptive for TB?				
6	# of contacts diagnosed with TB				
7	# of contacts eligible for TPT				
8	# of contacts initiated on TPT				
9	# of contacts enrolled on 3HR regimen?				
10	Treatment Outcome of contacts placed on 3HR-TPT?<br>1=Treatment completed<br>2= Lost to follow-up<br>3=Developed TB<br>4=Treatment interrupted<br>5=Refused Treatment				
